# Supplementary material for: Contribution of the IdyllaTM System to Improving the Therapeutic Care of Patients with NSCLC through Early Screening of EGFR Mutations
Source: Curr Oncol. 2021 Nov 3;28(6):4432–45. doi: 10.3390/curroncol28060376 (PMC8628756; doi:10.3390/curroncol28060376)
Supplement: Supplementary file 1 [file curroncol-28-00376-s001.zip › curroncol-1406705-SI.pdf]

Current Oncology
MDPI

Supplementary Materials

Contribution of the Idylla™ System to Improving the Therapeutic Care of Patients with NSCLC through Early Screening of *EGFR* Mutationss

Constance Petiteau [1,†], Gwladys Robinet-Zimmermann [2,†], Adèle Riot [3,†], Marine Dorbeau [2], Nicolas Richard [4,5], Cécile Blanc-Fournier [2], Frédéric Bibeau [3], Simon Deshayes [1], Emmanuel Bergot [1,6], Radj Gervais [7] and Guénaëlle Levallet [3,5,6,*]

[1] Department of Pulmonology & Thoracic Oncology, Centre Hospitalier Universitaire de Caen, F-14000 Caen, France;
petiteau.co@gmail.com (C.P.); deshayes-si@chu-caen.fr (S.D.); bergot-e@chu-caen.fr (E.B.)
[2] Department of Pathology, Centre François Baclesse, F-14000 Caen, France; gwladys.robinet.zimmermann@chu-rennes.fr (G.R.-Z.); m.dorbeau@baclesse.unicancer.fr (M.D.); c.blanc.fournier@baclesse.unicancer.fr (C.B.-F.)
[3] Department of Pathology, Centre Hospitalier Universitaire de Caen, F-14000 Caen, France; adele.riot@gmail.com (A.R.); fredbibo14@gmail.com (F.B.)
[4] Department of Genetics, Normandy University, UNICAEN, Caen University Hospital, EA 7450 BioTARGen, F-14000 Caen, France; richard-n@chu-caen.fr
[5] Federative Structure of Cyto-Molecular Oncogenetics (SF-MOCAE), Centre Hospitalier Universitaire de Caen, F-14000 Caen, France
[6] Normandy University, UNICAEN, CEA, CNRS, ISTCT Unit, GIP CYCERON, F-14000 Caen, France
[7] Department of Thoracic Oncology, Centre François Baclesse, F-14000 Caen, France; r.gervais@baclesse.unicancer.fr
* Correspondence: guenaelle.levallet@unicaen.fr
† Equal contribution.

Table S1. Mutations detected with Idylla™ EGFR Mutation cartridge.

Exon	Mutation	Frequency
	c.2156G>C, p.G719A	0.6%
	G719C mutation:	0.3%
**18**	• c.2155G>T, p.G719C	
	• c.2154_2155delinsTT, p.G719C	
	c.2155G>A, p.G719S	0.5%
	Exon 19 deletion:	
	• c.2238_2248delinsGC, Del9	
	• c.2239_2248delinsC, Del9	
	• c.2240_2248del, Del9	
	• c.2239_2247del, Del9	
	• c.2239_2251delinsC, Del12	
	• c.2240_2251del, Del12	
	• c.2235_2249del, Del15	
	• c.2236_2250del, Del15	
	• c.2239_2253del, Del15	
	• c.2240_2254del, Del15	
	• c.2238_2252del, Del15	
	• c.2237_2251del, Del15	
	• c.2235_2252delinsAAT, Del15	
	• c.2237_2252delinsT, Del15	
	• c.2234_2248del, Del15	
	• c.2236_2253delinsCTA, Del15	
	• c.2237_2253delinsTA, Del15	
**19**	• c.2235_2251delinsAG, Del15	48 %
	• c.2236_2253delinsCAA, Del15	
	• c.2230_2249delinsGTCAA, Del15	
	• c.2240_2257del, Del18	
	• c.2237_2255delins, Del18	
	• c.2239_2256del, Del18	
	• c.2236_2253del, Del18	
	• c.2239_2258delinsCA, Del18	
	• c.2237_2254del, Del18	
	• c.2238_2255del, Del18	
	• c.2237_2257delinsTCT, Del18	
	• c.2236_2255delinsAT, Del18	
	• c.2236_2256delinsATC, Del18	
	• c.2237_2256delinsTT, Del18	
	• c.2237_2256delinsTC, Del18	
	• c.2235_2255delinsGGT, Del18	
	• c.2238_2258del, Del21	
	• c.2236_2256del, Del21	
	• c.2253_2276del, Del24	
	c.2369C>T, p.T790M	
	c.2303G>T, p.S768I	
	Exon 20 insertion:	
	• c.2310_2311insGGT, InsG	<5%
**20**	• c.2307_2308insGCCAGCGTG, InsASV9	1.5 to 3%
	• c.2309_2310delinsCCAGCGTGGAT, InsASV11	4 to 9.2 %
	• c.2311_2312insGCGTGGACA, InsSVD	
	• c.2319_2320insCAC, InsH	
	L858R mutation:	43 %
	• c.2573T>G, p.L858R	
**21**	• c.2573_2574delinsGT, p.L858R	
	• c.2573_2574delinsGA, p.L858R	
	c.2582T>A, p.L861Q	2 %
